# Comparison of the Hepatoprotective Effects of Four Endemic Cirsium Species Extracts from Taiwan on CCl_4_-Induced Acute Liver Damage in C57BL/6 Mice

**DOI:** 10.3390/ijms19051329

**Published:** 2018-04-30

**Authors:** Zi-Wei Zhao, Jen-Chih Chang, Li-Wei Lin, Fan-Hsuan Tsai, Hung-Chi Chang, Chi-Rei Wu

**Affiliations:** 1 Department of Chinese Pharmaceutical Sciences and Chinese Medicine Resources, College of Pharmacy, China Medical University, Taichung 40402, Taiwan; wei111783@gmail.com; 2 Taichung Armed Forces General Hospital, Taichung 404, Taiwan; chang.jenchih@gmail.com; 3 The School of Chinese Medicines for Post-Baccalaureate, I-Shou University, Kaohsiung County 82445, Taiwan; lwlin@isu.edu.tw (L.-W.L.); asura0734@isu.edu.tw (F.-H.T.); 4 Department of Golden-Ager Industry Management, College of Management, Chaoyang University of Technology, Taichung 41394, Taiwan

**Keywords:** Cirsium species, phenylpropanoid glycosides, CCl_4_, liver damage, antioxidant, inflammation

## Abstract

Species of Cirsium (Asteraceae family) have been used in folk hepatoprotective medicine in Taiwan. We collected four Cirsium species—including the aerial part of *Cirsium arisanense* (CAH), the aerial part of *Cirsium kawakamii* (CKH), the flower part of *Cirsium japonicum* DC. var. *australe* (CJF), and Cirsii Herba (CH)—and then made extractions from them with 70% methanol. We compared the antioxidant contents and activities of these four Cirsium species extracts by a spectrophotometric method and high-performance liquid chromatography–photodiode array detector (HPLC-DAD). We further evaluated the hepatoprotective effects of these extracts on CCl_4_-induced acute liver damage in C57BL/6 mice. The present study found CAH possesses the highest antioxidant activity among the four Cirsium species, and these antioxidant activities are closely related to phenylpropanoid glycoside (PPG) contents. The extracts decreased serum ALT and AST levels elevated by injection with 0.2% CCl_4_. However, only CJF and CH decreased hepatic necrosis. Silibinin decreased serum alanine aminotransferase (ALT) and aspartate aminotransferase (AST) levels and hepatic necrosis caused by CCl_4_. CJF and CH restored the activities of hepatic antioxidant enzymes and decreased hepatic malondialdehyde (MDA) levels. CJF further restored the expression of hepatic antioxidant enzymes including Cu/Zn-superoxide dismutase (Cu/Zn-SOD), Mn-superoxide dismutase (Mn-SOD), and glutathione *S*-transferase (GST) proteins. HPLC chromatogram indicated that CKH, CJF, and CH contained silibinin diastereomers (α and β). Only CJF contained diosmetin. Hence, the hepatoprotective mechanism of CJF against CCl_4_-induced acute liver damage might be involved in restoring the activities and protein expression of the hepatic antioxidant defense system and inhibiting hepatic inflammation, and these hepatoprotective effects are related to the contents of silibinin diastereomers and diosmetin.

## 1. Introduction

The liver is an accessory digestive gland and plays a key role in the metabolism, detoxification, and secretory functions of the vertebrate body. A wide variety of viruses, drugs, and toxic chemicals can cause liver damage through their direct toxicity and/or metabolic toxic products. Drug-induced liver damage is responsible for 5% of all hospital admissions and 50% of patients suffered with acute liver failure [1]. It is known that carbon tetrachloride (CCl_4_) causes liver damage and hepatocyte apoptosis/necrosis in vitro and in vivo [2,3,4,5]. Liver damage from CCl_4_ is a common model used to measure the efficiency of many hepatoprotective drugs [6]. CCl_4_ is converted by hepatic cytochrome P450 2E1 (CYP2E1) into highly reactive radicals such as trichloromethyl (CCl3^•^) free radical and trichloromethylperoxy radical (CCl3OO^•^) [7]. Then, these radicals attack cellular macromolecules and cause lipid peroxidation, protein degradation, and DNA damage. The process is followed by the release of hepatic inflammatory cytokines such as interleukin-1β (IL-1β) and tumor necrosis factor-α (TNF-α), which leads to eventual damage, including hepatocellular necrosis [2,4,7].

Cirsii Herba (CH), listed in the Chinese Pharmacopoeia, is often mixed with *Cirsium japonicum* DC and *Cirsium setosum* (Willd.) MB in the traditional Chinese material market because they are similar in morphology and taxonomy. Chinese physicians usually use CH to treat hemorrhage, hypertension, and hepatitis. Recent pharmacological reports indicated that CH exhibits various pharmacological activities such as hemostatic, hepatoprotective, antidiabetic, anti-inflammatory, antibacterial, and sedative activities [8,9,10,11,12,13,14,15,16]. Due to its taxonomy (Asteraceae family) and morphology being similar to *Silybum marianum* (L.) Gaertn. (milk thistle), the Cirsium genus is commonly known as thistles and has been used to treat inflammation, diabetes, and hepatitis. *S. marianum* (L.) Gaertn has been used to protect liver injury caused by chemical and environmental toxins for a long time. Silymarin, the major active ingredient of *S. marianum*, includes flavonolignan isomers: 50–70% silibinin, isosilibinin, silydianin, and silychristin [17,18]. Silymarin also exerts hepatoprotective effects against alcoholic-induced liver disease, non-alcoholic fatty liver disease, and CCl_4_-induced liver damage [11,19,20]. Ten species of the Cirsium genus are described in the Taiwan flora [21], and *Cirsium arisanense* Kitam. (CA), *Cirsium kawakamii* Hayata (CK), and *Cirsium japonicum* DC. var. *australe* Kitam. (CJ) are known as “Formosan thistles” and commonly used as folk medicines in Taiwan. However, there are few scientific studies reporting the phytoconstituents and hepatoprotective activities of these three endemic Cirsium species from Taiwan. Therefore, this study compared the antioxidant activities of four Cirsium species against the ABTS (2,2′-azino-bis(3-ethylbenzothiazoline-6-sulphonic acid)) radical in vitro and their hepatoprotective effects against CCl_4_-induced acute liver damage in C57BL/6 mice. Silymarin and silibinin, the major ingredients of *Silybum marianum* (L.) Gaertn. and the most commonly known hepatoprotective agents, were used as positive controls. In addition, the mechanism of the hepatoprotective effect was studied regarding their potential antioxidant and anti-inflammatory properties. Moreover, previous phytochemical studies have revealed that flavonoids, lignans, phenylpropanoids, triterpenes, volatile oils, and steroids exist in CH, especially flavonoids [11,12,22,23,24,25]. Therefore, we also compared the phytoconstituents of four Cirsium species by spectrophotometric assay and high-performance liquid chromatography–photodiode array detector (HPLC-DAD).

## 2. Results

### 2.1. CCl_4_-Induced Acute Liver Damage in C57BL/6 Mice

#### 2.1.1. Serum Biochemical Levels of CCl_4_-Induced Acute Liver Damage in C57BL/6 Mice

**Figure 1:**
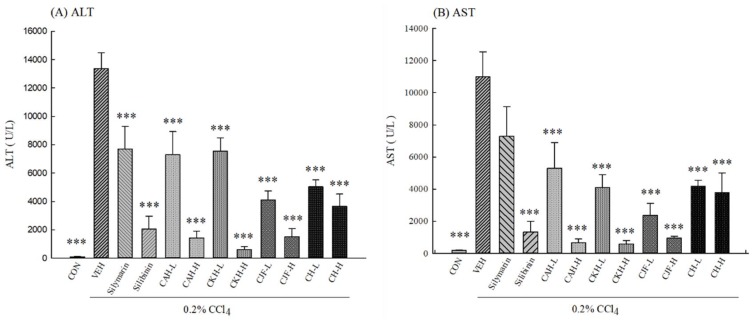
Effects of methanolic extracts of four Cirsium species (0.5, 5, 50 mg/kg), silymarin (100 mg/kg), and silibinin (25 mg/kg) on serum alanine aminotransferase (ALT) and aspartate aminotransferase (AST) levels of CCl_4_-indcued acute liver damage in C57BL/6 mice. (**A**) ALT; (**B**) AST. CAH: The aerial part of *C. arisanense* Kitam. CKH: The aerial part of *C. kawakamii* Hayata. CJF: The flower part of *C. japonicum* DC. var. *australe* Kitam. CH: Cirsii Herba. CAH-L, CAH-H, CKH-, CKH-H, CJF-L, CJF-H, CH-L, and CH-H were continuously administered for 7 days. One hour after last treatment, acute liver damage was induced by injection with 0.2% CCl_4_. Columns indicate mean ± SEM (*n* = 10). *** *p* < 0.001 compared with CCl_4_-indcued acute liver damage mice.

Serum alanine aminotransferase (ALT) and aspartate aminotransferase (AST) levels were used as biochemical markers for acute liver damage. One single-dose administration with 0.2% CCl_4_ resulted in a significant rise in the levels of serum ALT and AST compared with the control group in C57BL/6 mice (*p* < 0.001) (Figure 1). Pre-treatment with all doses of the four Cirsium species extracts for 7 days significantly prevented serum ALT and AST levels from being elevated by 0.2% CCl_4_ (*p* < 0.001) (Figure 1). Silibinin (positive control) at a dose of 25 mg/kg also prevented the elevation of serum ALT and AST levels, but silymarin (positive control) at a dose of 100 mg/kg only prevented the elevation of serum ALT levels (*p* < 0.001) (Figure 1).

#### 2.1.2. Hepatic Histopathology of CCl_4_-Induced Acute Liver Damage in C57BL/6 Mice

**Figure 2:**
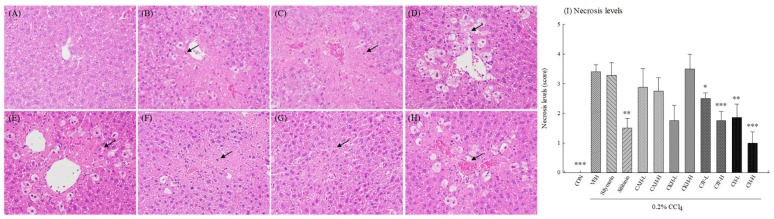
Effects of methanolic extracts of four Cirsium species (0.5, 5, 50 mg/kg), silymarin (100 mg/kg), and silibinin (25 mg/kg) on hepatic histopathology of CCl_4_-indcued acute liver damage in C57BL/6 mice. (**A**) Hematoxylin and eosin (H&E) stain of control group; (**B**) H&E stain of CCl_4_ group; (**C**) H&E stain of silymarin group; (**D**) H&E stain of silibinin group; (**E**) H&E stain of CAH-H group; (**F**) H&E stain of CKH-H group; (**G**) H&E stain of CJF-H; (**H**) H&E stain of CH-H; (**I**) Necrosis levels. CAH: The aerial part of *C. arisanense* Kitam. CKH: The aerial part of *C. kawakamii* Hayata. CJF: The flower part of *C. japonicum* DC. var. *australe* Kitam. CH: Cirsii Herba. CAH-L, CAH-H, CKH-, CKH-H, CJF-L, CJF-H, CH-L, and CH-H were continuously administered for 7 days. One hour after last treatment, acute liver damage was induced by injection with 0.2% CCl_4_. Representative images (**A**–**H**) of hematoxylin and eosin (H&E) with 400× amplification. The black arrows indicate hepatocyte necrosis. Columns (**I**) indicate mean ± SEM (*n* = 10). * *p* < 0.05, ** *p* < 0.01, *** *p* < 0.001 compared with CCl_4_-indcued acute liver damage mice.

Hepatic histopathology provided supporting evidence for the serum biochemical analysis. The hepatic histopathology in control groups showed normal hepatocytes (Figure 2A). One single-dose administration with 0.2% CCl_4_ caused a loss of hepatic architecture including vacuole formation, inflammatory infiltration, and necrosis in C57BL/6 mice (Figure 2B). Silibinin, CJF, and CH had different degrees of improving effects on CCl_4_-induced histological changes (Figure 2C–H). There were higher hepatocyte necrosis levels in one single-dose administration with 0.2% CCl_4_ (*p* < 0.001) (Figure 2I). Only CJF-L, CJF-H, CH-L, and CH-H inhibited the liver-damage phenomenon induced by CCl_4_ (*p* < 0.05, 0.001, 0.01, 0.001, respectively) (Figure 2I). Silymarin, CAH, and CKH did not decrease hepatocyte necrosis levels induced by CCl_4_ (*p* > 0.05) (Figure 2I).

#### 2.1.3. Hepatic Antioxidant Activities and MDA Levels of CCl_4_-Induced Acute Liver Damage in C57BL/6 Mice

**Figure 3:**
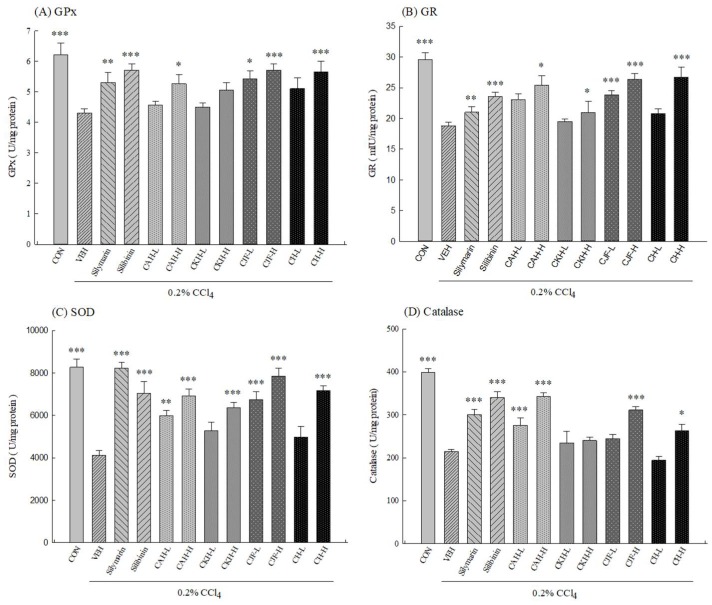
Effects of methanolic extracts of four Cirsium species (0.5, 5, 50 mg/kg), silymarin (100 mg/kg) and silibinin (25 mg/kg) on hepatic antioxidant enzyme activities of CCl_4_-induced acute liver damage in C57BL/6 mice. (**A**) glutathione peroxidase (GPx); (**B**) glutathione reductase (GR); (**C**) superoxide dismutase (SOD); (**D**) catalase. CAH: The aerial part of *C. arisanense* Kitam. CKH: The aerial part of *C. kawakamii* Hayata. CJF: The flower part of *C. japonicum* DC. var. *australe* Kitam. CH: Cirsii Herba. CAH-L, CAH-H, CKH-, CKH-H, CJF-L, CJF-H, CH-L, and CH-H were continuously administered for 7 days. One hour after last treatment, acute liver damage was induced by injection with 0.2% CCl_4_. Columns indicate mean ± SEM (*n* = 10). * *p* < 0.05, ** *p* < 0.01, *** *p* < 0.001 compared with CCl_4_-indcued acute liver damage mice.

**Figure 4:**
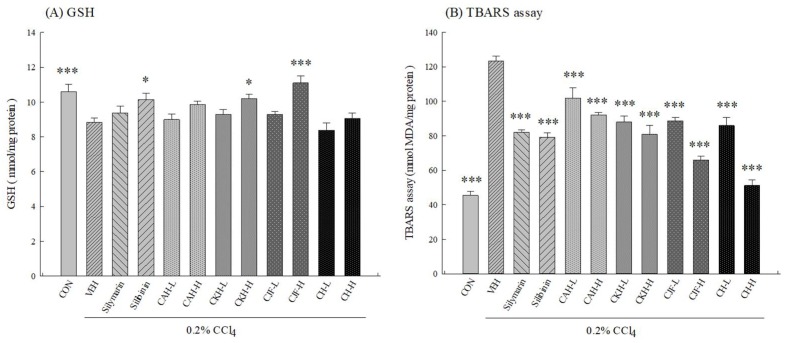
Effects of methanolic extracts of four Cirsium species (0.5, 5, 50 mg/kg), silymarin (100 mg/kg), and silibinin (25 mg/kg) on hepatic glutathione (GSH) and malondialdehyde (MDA) levels of CCl_4_-indcued acute liver damage in C57BL/6 mice. (**A**) GSH; (**B**) MDA. TBARS: thiobarbituric acid reactive substances. CAH: The aerial part of *C. arisanense* Kitam. CKH: The aerial part of *C. kawakamii* Hayata. CJF: The flower part of *C. japonicum* DC. var. *australe* Kitam. CH: Cirsii Herba. CAH-L, CAH-H, CKH-, CKH-H, CJF-L, CJF-H, CH-L, and CH-H were continuously administered for 7 days. One hour after last treatment, acute liver damage was induced by injection with 0.2% CCl_4_. Columns indicate mean ± SEM (*n* = 10). * *p* < 0.05, *** *p* < 0.001 compared with CCl_4_-indcued acute liver damage mice.

To clarify the role of the antioxidative defense system on the hepatoprotective effects of four Cirsium species extracts against CCl_4_-induced acute liver damage in C57BL/6 mice, we measured the activities of the hepatic antioxidant defense system, including the level of glutathione (GSH), the activities of related antioxidant enzymes, and the level of oxidative damage markers such as malondialdehyde (MDA). One single-dose intraperitoneal injection with 0.2% CCl_4_ decreased the activities of hepatic antioxidant enzymes including superoxide dismutase (SOD), glutathione peroxidase (GPx), glutathione reductase (GR), and catalase in rats (*p* < 0.001) (Figure 3). We further found one single-dose intraperitoneal injection with 0.2% CCl_4_ also decreased hepatic GSH levels but increased hepatic MDA levels in C57BL/6 mice (*p* < 0.001) (Figure 4). CAH-L and CAH-H could restore hepatic SOD and catalase activities and decrease hepatic MDA levels (*p* < 0.01, 0.001), but only CAH-H restored hepatic GPx and GR activities (*p* < 0.05) (Figure 3 and Figure 4). Only CKH-H could restore hepatic SOD and GR activities and GSH levels (*p* < 0.05, 0.001), but CKH-L and CKH-H decreased hepatic MDA levels (*p* < 0.001) (Figure 3 and Figure 4). CJF-L and CJF-H could restore hepatic GPx, GR, and SOD activities and decrease hepatic MDA levels, but only CJF-H restored hepatic catalase activity and GSH levels (*p* < 0.001) (Figure 3 and Figure 4). Only CH-H could restore the activities of hepatic antioxidant enzymes (*p* < 0.05, 0.001), but CKH-L and CKH-H decreased hepatic MDA levels (*p* < 0.001) (Figure 3 and Figure 4). Silymarin (100 mg/kg) and silibinin (25 mg/kg) restored the activities of all hepatic antioxidant enzymes and decreased hepatic MDA levels (*p* < 0.01, 0.001) (Figure 3 and Figure 4). However, only silibinin could restore hepatic GSH level decreased by CCl_4_ (*p* < 0.05) (Figure 4).

#### 2.1.4. Hepatic Cytokine Levels of CCl_4_-Induced Acute Liver Damage in C57BL/6 Mice

**Figure 5:**
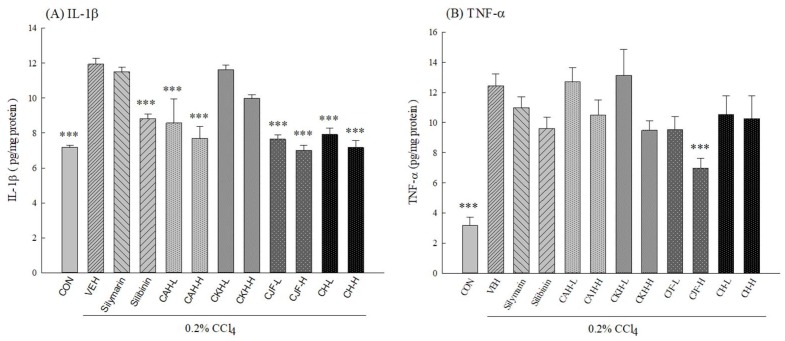
Effects of methanolic extracts of four Cirsium species (0.5, 5, 50 mg/kg), silymarin (100 mg/kg), and silibinin (25 mg/kg) on hepatic cytokine levels of CCl_4_-indcued acute liver damage in C57BL/6 mice. (**A**) interleukin-1β (IL-1β); (**B**) tumor necrosis factor-α (TNF-α). CAH: The aerial part of *C. arisanense* Kitam. CKH: The aerial part of *C. kawakamii* Hayata. CJF: The flower part of *C. japonicum* DC. var. *australe* Kitam. CH: Cirsii Herba. CAH-L, CAH-H, CKH-L, CKH-H, CJF-L, CJF-H, CH-L, and CH-H were continuously administered for 7 days. One hour after last treatment, acute liver damage was induced by injection with 0.2% CCl_4_. Columns indicate mean ± SEM (*n* = 10). *** *p* < 0.001 compared with CCl_4_-indcued acute liver damage mice.

To clarify the anti-inflammatory mechanism on the hepatoprotective effects of four Cirsium species extracts against CCl_4_-induced acute liver damage in C57BL/6J mice, we measured the levels of hepatic cytokines, including IL-1β and TNF-α. One single-dose intraperitoneal injection with 0.2% CCl_4_ elevated the levels of hepatic cytokines in C57BL/6 mice (*p* < 0.001) (Figure 5). CAH, CJF, and CH-H decreased hepatic IL-1β levels (*p* < 0.001) (Figure 5). Only CJF-H decreased hepatic TNF-α levels (*p* < 0.001) (Figure 5). Silibinin (25 mg/kg) decreased hepatic IL-1β levels (*p* < 0.001) (Figure 5). However, silymarin (100 mg/kg) and CKH did not decrease the levels of hepatic cytokines elevated by CCl_4_ (*p* > 0.05) (Figure 5).

#### 2.1.5. Antioxidant Protein Expression of CCl_4_-Induced Acute Liver Damage in C57BL/6 Mice

**Figure 6:**
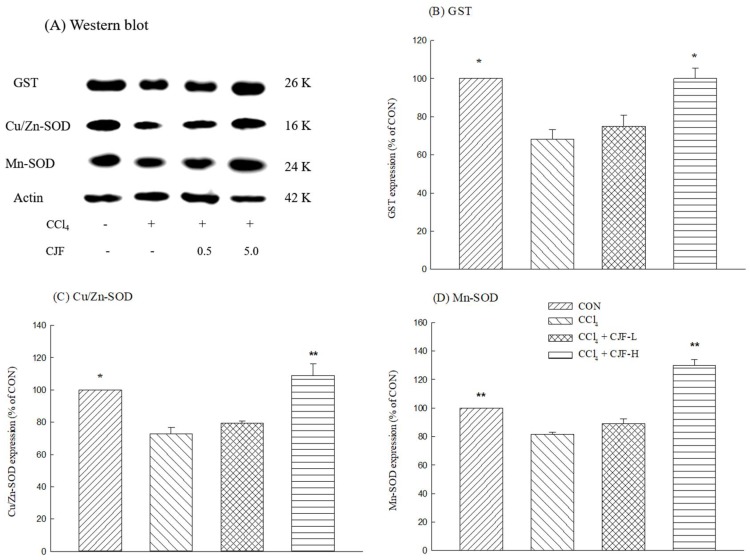
Effects of methanolic extracts of the flower part of *C. japonicum* DC. var. *australe* Kitam. (CJF; 0.5, 5 mg/kg) on the GST, Cu/Zn-SOD, and Mn-SOD expression of CCl_4_-indcued acute liver damage in C57BL/6 mice. (**A**) Western blot; (**B**) GST; (C) Cu/Zn-SDO; (D) Mn-SOD. CJF-L and CJF-H were continuously administered for 7 days. One hour after last treatment, acute liver damage was induced by injection with 0.2% CCl_4_. Columns indicate mean ± SEM (*n* = 4). * *p* < 0.05, ** *p* < 0.01 compared with CCl_4_-indcued acute liver damage mice.

The protein immunoblot assay is shown in Figure 6A. One single-dose intraperitoneal injection with 0.2% CCl_4_ decreased hepatic GST, Cu/Zn-SOD, and Mn-SOD expression levels in C57BL/6 mice (*p* < 0.05, 0.01) (Figure 6B–D). Only CJF-H restored hepatic GST, Cu/Zn-SOD, and Mn-SOD expression levels downregulated by CCl_4_ (*p* < 0.05, 0.01, respectively) (Figure 6B–D).

### 2.2. Antioxidant Ingredient Contents and Activities of Four Cirsium Species Extracts

#### 2.2.1. Total Phenolics (TPs) Contents, Total Phenylpropanoid Glycosides (PPGs) Contents, and HPLC Analysis of Four Cirsium Species Extracts

**Figure 7:**
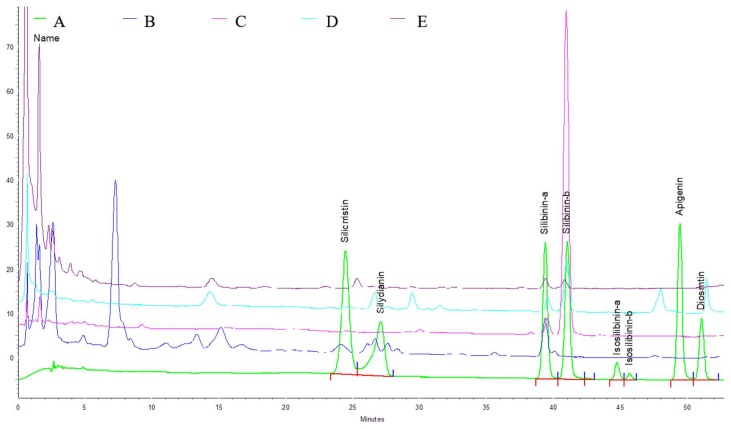
High-performance liquid chromatography (HPLC) chromatograms of methanolic extracts of four Cirsium species at 280 nm. Trace: (**A**) Standard, (**B**) CAH: The aerial part of *C. arisanense* Kitam., (**C**) CKH: The aerial part of *C. kawakamii* Hayata. (**D**) CJF: The flower part of *C. japonicum* DC. var. *australe* Kitam. (**E**) CH: Cirsii Herba.

The contents of TPs and PPGs in four Cirsium species extracts are shown in Table 1. There are the higher contents of TPs and PPGs in CAH compared with the other three Cirsium species extracts. The order of the TPs contents of the other three Cirsium species extracts is CKH > CJF > CH. However, the order of the PPGs contents for the other three Cirsium species extracts is CJF > CH > CKH. Furthermore, the phytoconstituents of the four Cirsium species extracts were assayed using HPLC. Their chromatographs are shown in Figure 7. The certain phytoconstituent peak zones differ among the four Cirsium species extracts. The calibration curves for apigenin, diosmetin, silibinin α, silibinin β, silydianin, silychristin, isosilibinin α, and isosilibinin β were drawn in the concentration range of 2.5–20 μg/mL. The correlation coefficients of the calibration plots were equal to 0.992–0.994, indicating good linearity in four cases. The methanolic extracts of the four Cirsium species contained silibinin α, and CAH had the highest content among the four Cirsium species. CKH, CJF, and CH contained silibinin β. Only CAH and CJF contained silydianin. Diosmetin only existed in CJF (Table 1).

#### 2.2.2. Antioxidant Activities of Four Cirsium Species Extracts

**Figure 8:**
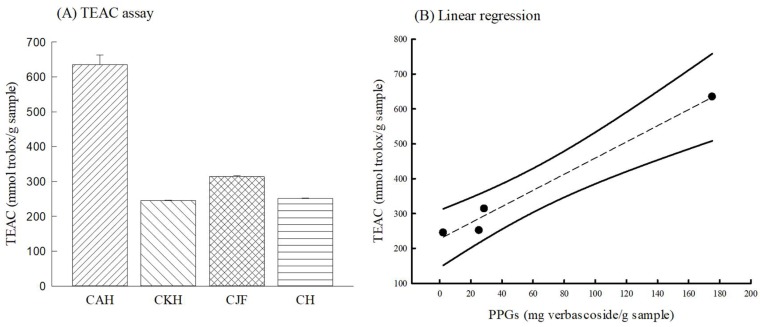
Antioxidant activity of methanolic extracts of four Cirsium species. (**A**) Trolox equivalent antioxidant capacity (TEAC); (**B**) the relationship between TEAC and PPGs content. Columns indicate mean ± SD (*n* = 3). CAH: The aerial part of *C. arisanense* Kitam. CKH: The aerial part of *C. kawakamii* Hayata. CJF: The flower part of *C. japonicum* DC. var. *australe* Kitam. CH: Cirsii Herba.

The Trolox Equivalent Antioxidant Capacity (TEAC) assay is a simple and rapid method that is commonly used to assess total antioxidant capacity related to the scavenging 2,2′-azino-bis(3-ethylbenzothiazoline-6-sulphonic acid) (ABTS) radical and reactive oxygen species (ROS) in vitro. The total antioxidant capacities of the four Cirsium species extracts in the TEAC assay are shown in Figure 8A and expressed as a Trolox equivalent (mmol trolox/g sample). CAH had the highest antioxidant capacity against ABTS radical compared with the other three Cirsium species. Then, we further examined the relationship between TEAC and the contents of antioxidant phytoconstituents by Pearson correlation analysis. Only the PPGs content was positively and highly correlated with TEAC (*r* = 0.99) (Figure 8B).

## 3. Discussion

The Cirsium genus is similar to *Silybum marianum* (L.) Gaertn based on the taxonomy, morphology, and folk medical use. They are commonly known as thistles and have been used to treat inflammation and hepatitis. *Silybum marianum* and its ingredient silymarin also exert hepatoprotective effects against alcohol-induced liver disease, non-alcoholic fatty liver disease, and CCl_4_-induced liver damage [11,19,20]. CCl_4_ is the oldest and most widely used hepatic toxin, and one single-dose injection with CCl_4_ in rodents is often used as an acute liver damage model for screening hepatoprotective drugs [6]. CCl_4_-induced liver damage is mainly due to the lipid peroxidation of hepatocyte membranes by free radicals derived from CCl_4_ metabolites. Membrane destruction of hepatocytes leads to the release of hepatic enzymes such as ALT and AST into blood circulation. Consequently, serum ALT and AST levels are used as biochemical markers of liver damage [26]. In this study, an acute CCl_4_ liver-damage model was used to compare the hepatoprotective efficacy of four Cirsium species extracts, with silymarin and silibinin being used as positive controls. We found that one single-dose injection with CCl_4_ drastically elevated serum ALT and AST levels in C57BL/6 mice, which indicates CCl_4_-induced acute liver damage. The present results demonstrated that all four Cirsium species extracts at any dose could decrease serum ALT and AST levels elevated by CCl_4_. Further, from the serum biochemical analysis by hepatic histopathological stain, we found CCl_4_ caused dysfunction of hepatic cellular structures, including vacuole formation, inflammatory infiltration, and necrosis in C57BL/6 mice. However, there was minimal disruption of the hepatic cellular structure and less necrosis against CCl_4_-induced acute liver injury when pretreatment with only CJF and CH (at any dose) and silibinin for 7 days, compared with the CCl_4_ group. Previous reports indicated that CH (*C. japonicum* DC or *C. setosum*) exhibited hepatoprotective effects against CCl_4_-induced hepatotoxicity in L02 cells or HL-7702 cells [11,12]. Ku et al. reported that the root, but not aerial part, of CA possessed hepatoprotective properties against tacrine-induced hepatotoxicity in Hep 3B cells and C57BL/6 mice [27]. Therefore, our present results from serum biochemical and histopathological data demonstrate that only CJF and CH, among the four Cirsium species, possessed convincing hepatoprotective potency against CCl_4_-induced acute liver damage, although all four endemic Cirsium species decreased serum ALT and AST levels. Two positive controls, silymarin and silibinin, could decrease serum ALT and AST levels elevated by CCl_4_, but only silibinin prevented dysfunction of the hepatic cellular structure. Wu et al. indicated that silymarin did not decrease serum ALT and AST levels and histopathological alteration caused by CCl_4_ in mice [28], although many reports have found that silymarin at 200–800 mg/kg decreased liver damage caused by acetaminophen, ethanol, and CCl_4_ [29,30,31]. Hence, our results confirmed other reports [32] that silibinin is a major active constituent of silymarin, and at 20–50 mg/kg prevented liver damage caused by acetaminophen, ethanol, and CCl_4_.

Experimental and clinical reports indicate that oxidative stress plays a critical role in the development of drug-induced liver damage [33]. CCl_4_-induced liver damage is mostly due to reactive free-radical generation from the dehalogenation of CCl_4_ by CYP2E1. These reactive free radicals, including trichloromethyl radical (CCl3•) and trichloromethylperoxy radical (CCl3OO•), cause the depletion of hepatic antioxidant status and the exacerbation of hepatic lipid peroxidation [7]. The major hepatic antioxidant defense system against free radicals includes SOD, catalase, and GSH redox cycle. SOD, the first enzyme involved in the antioxidant defense system, scavenges superoxide anions, which are produced from the mitochondria electron transfer chain. When superoxide anions are transformed into H_2_O_2_ by SOD, catalase then continuously detoxifies it to H_2_O. The GSH redox cycle, mainly including GSH, GPx, and GR, modulates the redox-mediated responses of hepatic cells induced by external or intracellular stimuli. GSH, a cytosolic tripeptide, is the major non-enzymatic regulator of intracellular redox homeostasis. GSH scavenges hydroxyl radicals directly and is as a cofactor in detoxifying hydrogen peroxide, lipid peroxides, and alkyl peroxides by catalytic action of the detoxifying enzyme GPx. GPx, a selenocysteine-containing enzyme, reduces lipid hydroperoxides to their corresponding alcohols and hydrogen peroxide to water in the liver. GR is essential for the glutathione redox cycle and maintains adequate levels of reduced cellular GSH, which catalyzes the reduction of oxidized GSH to reduce GSH. In this regard, the enhancement of the hepatic antioxidant system capacity may be an effective therapeutic strategy for the alleviation and treatment of liver damage [33,34]. Our present data showed that one single-dose injection with CCl_4_ induced significant depletion of GSH levels, dysfunction of SOD, catalase, GR, and GPX, and enhanced lipid peroxidation in liver homogenates. Hence, the results confirmed that CCl_4_ decreases the function of antioxidant enzymes and the GSH redox cycle to alter the intracellular redox status, causing oxidative stress and enhancing lipid peroxidation in the liver. Here, our results showed that pretreatment with CJF or silibinin for 7 days significantly restored GSH levels and the activities of antioxidant enzymes, including SOD, catalase, GPx, and GR, and reduced MDA levels in mice liver homogenate. Pretreatment with CH also restored the activities of antioxidant enzymes but not the GSH redox cycle. However, CH still reduced hepatic MDA levels. Previous studies have shown that *C. japonicum* DC can decrease hepatic lipid peroxidation along with an increase in hepatic content of reduced glutathione in ethanol-treated rats. CJF, similar to silibinin, might play a role in restoring hepatic redox capacity and antioxidant enzyme activities, resulting in a protective effect against CCl_4_-induced acute liver damage. Other Cirsium species are not so comprehensive in restoring hepatic redox capacity and antioxidant enzyme activities against CCl_4_-induced acute liver damage. 

Moreover, GST is a phase II metabolic enzyme. It increases cellular GSH levels and protects cells against oxidative stress through conjugating free radicals with GSH [35]. SOD consists of three isoforms in mammals: the cytoplasmic Cu/Zn-SOD (SOD1), the mitochondrial Mn-SOD (SOD2), and the extracellular Cu/Zn-SOD (SOD3). Cu/Zn-SOD (SOD1) is mainly localized in the cytosol, with a smaller fraction in the intermembrane space of mitochondria. Cu/Zn-SOD (SOD1) comprises 90% of the total SOD and has great physiological significance and therapeutic potential. Mn-SOD is a manganese (Mn) containing enzyme and is localized in the mitochondrial matrix. The essential role of Mn-SOD is in maintaining mitochondrial function [36]. Hence, we further explored the role of GST, Cu/Zn-SOD, and Mn-SOD proteins on the hepatoprotective effects of CJF against CCl_4_-induced acute liver damage in C57BL/6 mice. CCl_4_ decreased the expression of GST, Cu/Zn-SOD, and Mn-SOD proteins in liver homogenates. CJF, at a high dose, can restore the expression of GST, Cu/Zn-SOD, and Mn-SOD proteins downregulated by CCl_4_. Therefore, the hepatoprotective mechanism of CJF is related to restoring the activities of the hepatic antioxidant defense system through reversing the GSH redox cycle and the activities and expression of antioxidant enzymes.

During the pathological events of acute liver damage with CCl_4_, inflammatory processes play a crucial role. Resident macrophages in the liver, known as Kupffer cells, are activated and rapidly release pro-inflammatory cytokines such as TNF-α and IL-1β. These pro-inflammatory cytokines play an important role in a complex network involved in the regulation of inflammatory responses. The increase in TNF-α and IL-1β levels is correlated with the histopathological evidence of hepatic necrosis and the increase in serum ALT and AST levels. Therefore, inhibiting the activity of macrophages and the release of pro-inflammatory cytokines provides an excellent therapeutic strategy to alleviate liver inflammation and damage [37]. Furthermore, we also investigated the effects of four Cirsium species on the levels of hepatic pro-inflammatory cytokines, such as TNF-α and IL-1β, in CCl_4_-induced acute liver damage. This study confirmed that CCl_4_ administration caused the increase in hepatic TNF-α and IL-1β levels in C57BL/6 mice. Only CJF markedly reduced the increased TNF-α and IL-1β levels caused by CCl_4_ in mice liver homogenates. CH and silibinin only decreased the high IL-1β levels caused by CCl_4_. Thus, the present results demonstrate that CJF has hepatoprotective potency against CCl_4_-induced acute liver damage by its antioxidative and anti-inflammatory features via reversal of the hepatic antioxidant defense system and suppression of pro-inflammatory cytokine expressions.

Finally, we compared antioxidant activities and the contents of four Cirsium species in vitro because their hepatoprotective effects are partially related to their antioxidant activities. In this study, CAH possessed the highest scavenging activity against the ABTS radical. CAH also had the highest TPs and PPGs content. According to the Pearson’s analysis, there is a close correlation between antioxidant (scavenging ABTS radical) activities and PPGs contents. However, this result is inconsistent with the hepatoprotective result. We further assayed the phytoconstituents of the four Cirsium species by HPLC-DAD. There are different HPLC fingerprints among the four Cirsium species to distinguish them from this result. CKH, CJF, and CH contained silibinin diastereomers (α and β), with their order being CKH > CJF > CH. CJF still contained silydianin and diosmetin. CAH only contained silydianin and silibinin α, inconsistent with other reports stating that no related compounds existed in CAH [27]. This study provides the first reported results concerning the phytoconstituents of CAH, CKH, and CJF. The results regarding the phytoconstituents of CH are consistent with other reports [11]. However, we found that silibinin diastereomers might not be the active ingredients of CKH, CJF, and CH associated with their hepatoprotective effects against CCl_4_ because there is no correlation between the contents of silibinin diastereomers and hepatoprotective activity. The hepatoprotective phytoconstituents of CH might include flavonoids, such as silibinin diastereomers and phenylethanoid glycosides, because the hepatoprotective ingredients of *C. japonicum* are flavonoids and the hepatoprotective ingredients of *C. setosum* are phenylethanoid glycosides [11,12]. The efficacy of CJF, which showed the best hepatoprotective effects among the four Cirsium species, might be due to silibinin diastereomers and diosmetin because diosmetin could inhibit CYP 2E1 [38] and it possesses antioxidant and anti-inflammatory activities against endotoxin-induced acute liver injury [39]. Therefore, the hepatoprotective phytoconstituents of CH against CCl_4_-induced acute liver damage must be clarified. The synergistic effects of silibinin diastereomers and diosmetin against CCl_4_-induced acute liver damage deserve to be investigated in the future. The hepatoprotective effects of CJF against CCl_4_-induced hepatic cirrhosis must also be investigated.

## 4. Materials and Methods

### 4.1. Collection and Preparation of Plant Materials

Three Cirsium species materials including the aerial part of CA (CAH), the aerial part of CK (CKH), and the flower part of CJ (CJF), were identified and provided by Hung-Chi Chang. CH was obtained from the Herb Garden of China Medical University, Taichung City. Four Cirsium species materials were extracted with 10 times volume of 70% methanol, and the resulting extracts were concentrated under reduced pressure to obtain the four Cirsium species methanolic extracts. To assess phytoconstituents and ROS-scavenging activities, the extracts were dissolved in distilled water. To compare the hepatoprotective effects on CCl_4_-induced acute liver damage, the four Cirsium species methanolic extracts were prepared using 0.5% carboxymethylcellulose (CMC).

### 4.2. CCl_4_-Induced Acute Liver Damage in C57BL/6 Mice

#### 4.2.1. Animals

Male C57BL/6 mice (20–25 g) were obtained from BioLASCO Taiwan Co., Ltd. They were housed in groups of six, chosen at random, in wire-mesh cages (39 cm × 26 cm × 21 cm) in a temperature (23 ± 1 °C) and humidity (60%) regulated environment with a 12 h–12 h light/dark cycle (light phase: 8:00 a.m. to 8:00 p.m.). The Institutional Animal Care and Use Committee of China Medical University approved the experimental protocol (No. CMUIACUC-2017392), and mice were cared for according to the Guiding Principles for the Care and Use of Laboratory Animals. After one week of acclimatization, the mice were used in CCl_4_-induced acute liver damage.

#### 4.2.2. CCl_4_-Induced Acute Liver Damage in C57BL/6 Mice

The mice were randomly divided into 12 groups of 10 mice each. In the control group and CCl_4_ group, mice were orally given 0.5% CMC (0.1 mL/10 g body weight) daily for 7 days. In the treatment groups, other mice were separated into 10 groups: (1) CCl_4_ + silymarin: this group served as a positive control. Mice were orally given silymarin (100 mg/kg) daily for 7 days; (2) CCl_4_ + silibinin: this group also served as a positive control. Mice were orally given silibinin (25 mg/kg) daily for 7 days; (3) CCl_4_ + CAH-L: CAH-L (5 mg/kg) was orally given daily for 7 days; (4) CCl_4_ + CAH-H: CAH-H (50 mg/kg) was orally given daily for 7 days; (5) CCl_4_ + CKH-L: CKH-L (5 mg/kg) was orally given daily for 7 days; (6) CCl_4_ + CKH-H: CKH-H (50 mg/kg) was orally given daily for 7 days; (7) CCl_4_ + CJF-L: CJF-L (0.5 mg/kg) was orally given daily for 7 days; (8) CCl_4_ + CJF-H: CJF-H (5 mg/kg) was orally given daily for 7 days; (9) CCl_4_ + CH-L: CH-L (5 mg/kg) was orally given daily for 7 days; (10) CCl_4_ + CH-H: CH-H (50 mg/kg) was orally given daily for 7 days. On the seventh day, all mice except those in the control groups were intraperitoneally given 0.2% CCl_4_ (dissolved in olive oil, 0.1 mL/10 g body weight) 1 h after the last administration, while the control group received olive oil [40]. All mice were fasted for 24 h and subsequently blood was collected via retroorbital sinus plexus under isoflurane anesthesia. Then, all mice were sacrificed, and their livers were dissected and used for histopathological (formalin fixed) and biochemical (frozen −80 °C) studies.

#### 4.2.3. Assessment of Liver Function

After blood collection, serum was separated by centrifugation at 3000 rpm at room temperature (RT) for 30 min and was kept at −20 °C for further biochemical analysis. Serum ALT and AST values were measured with commercially available Roche diagnostic kits.

#### 4.2.4. Hepatic Antioxidant Defense System and MDA Levels

All hepatic tissues were homogenized in 9 vol ice-cold phosphate buffered saline. Homogenates were centrifuged at 12,000 rpm for 15 min at 4 °C, and the aliquots of supernatants were separated and stored at –80 °C until use. The aliquots of supernatants were used to determine antioxidant enzyme activities for catalase, GPx, GR, and SOD, and the levels of MDA and GSH. The activities of antioxidant enzymes and the levels of MDA and GSH were measured with a spectrophotometric microplate reader, as we previously reported [41]. First, catalase activity was determined with the decrease in the absorbance of amplex red at 560 nm. SOD activity was measured kinetically with the production of nitroblue tetrazolium, the absorbance of which is at 560 nm. The activities of GPx and GR were measured by a Cayman assay kit. SOD and catalase was expressed as U/mg of protein. GPx activities were expressed as U/mg of protein. GR activities were expressed as mIU/mg of protein. GSH standard solution or the supernatant solution (20 μg/50 μL) was pipetted into each well of a 96-well plate. The reaction solution, including 660 μM DTNB (5,5′-dithio-bis-(2-nitrobenzoic acid)), 900 μM NADPH (β-nicotinamide adenine dinucleotide phosphate), and 4.5 U/mL GR, was added to each well and then recorded at 405 nm for 5 min in a microplate reader. GSH levels were expressed as mmol/mg of protein. The thiobarbituric acid reactive substances (TBARS) assay was used to measure MDA levels. MDA standard solution or the supernatant solution was pipetted into 1.5 mL tubes, and a thiobarbituric acid (TBA) test was performed. Next, the absorbance of the above reaction solution was determined at 532 nm. TBARS assays were expressed as MDA equivalents (mmol MDA/mg of protein).

#### 4.2.5. Hepatic Cytokines Levels

Hepatic tissues were homogenized with protease inhibitor solution (0.4 M NaCl, 0.05% Tween 20, 0.5% bovine serum albumin, 0.1 mM phenylmethylsulfonylfluoride, 0.1 mM benzethonium chloride, 10 mM EDTA, 10 μg/mL aprotinin). Homogenates were centrifuged at 12,000 rpm at 4 °C, aliquoted, and stored at −80 °C until analysis. IL-1β and TNF-α protein levels were assessed using ELISA kits (R&D Systems, Abingdon, UK) according to the manufacturer protocol. The levels of IL-1β and TNF-α were expressed as pg/mg of protein.

#### 4.2.6. Histopathological Stain

A portion of the left lobe of the liver was preserved in 10% neutral formalin solution, processed, and paraffin embedded, as per the standard protocol. Sections of 4 μm in thickness were cut, deparaffinized, dehydrated, and stained with hematoxylin and eosin (H&E) for the estimation of hepatocyte necrosis and vacuolization. Morphological changes were observed including cell gross necrosis, ballooning degeneration, and inflammatory infiltration.

#### 4.2.7. Western Blot

Hepatic tissues were subjected to Western blot analyses to determinate the protein levels of Cu/Zn-SOD, Mn-SOD, and GST. Briefly, the hepatic tissues were cut into small pieces and homogenized in 9× cold lysis buffer (20 mM HEPES pH 7.0, 10 mM KCl, 0.5% NP-40) with a tissue grinder. The homogenate was incubated for 10 min and centrifuged at 12,000 rpm for 20 min to obtain the cytoplasmic supernatant. The aliquots of cytoplasmic supernatants were stored at –80 °C until use. The protein concentration was quantified using a Bradford protein assay kit (Bio-Rad Ltd. Inc., Hercules, CA, USA) and followed by electrophoretic separation through SDS-PAGE. After transferring the protein samples to PVDF membranes, the samples were blocked with 5% non-fat dry milk and 0.1% tween-20 in tris-buffered saline at room temperature for 1 h. Then, the membranes were incubated with primary antibodies against Cu/Zn-SOD, Mn-SOD, and GST (Santa Cruz Biotechnology, Dallas, TX, USA) overnight at 4 °C and subsequently incubated with horseradish peroxidase-conjugated goat anti-rabbit or goat anti-mouse Immunoglobulin G (IgG). The images were scanned using an LAS-4000 mini imaging system (Fujifilm, Kanagawa, Japan), and the optical density data was analyzed using MultiGauge v3.0 software (Fujifilm). For the Western blot analyses, β-actin (Proteintech, Rosemont, IL, USA) served as an internal control.

### 4.3. Measurement of Antioxidant Phytoconstituent Contents and Activities

#### 4.3.1. Measurement of Phytoconstituents Using a Spectrophotometric Reader

The contents of all phytochemicals, including TPs and PPGs, were assayed using a 96-well BioTek PowerWave™ 340 microtiter spectrophotometric reader (BioTek Inc., Winooski, VT, USA). The measured method for TPs contents is based on forming blue-colored products through a redox reaction with Folin-Ciocalteu’s reagent and measuring its absorbance at 725 nm. The TPs contents of the four Cirsium species methanolic extracts were expressed as milligrams of catechin equivalents per gram of the four extracts [28]. The measured method for PPGs contents is based on forming colored products through PPGs with Arnow reagent (containing 5% (*w*/*v*) sodium nitrate and 5% sodium molybdate) and measuring its absorbance at 525 nm. The PPGs contents of the four extracts were expressed as milligrams of verbascoside equivalents per gram of the extracts [28].

#### 4.3.2. Determination of Phytoconstituents Using HPLC-DAD

The extracts were dissolved in methanol and then filtered using a 0.22 μm filter. Stock solutions of the standards including apigenin, diosmetin, silibinin α, silibinin β, silydianin, silychristin, isosilibinin α, and isosilibinin β were prepared in methanol to the final concentration 10 mg/mL. Working solutions at 2.5–20 μg/mL were prepared freshly every day by dilution of the standard solutions with methanol. All standard and sample solutions were injected into 10 μL in triplicate. The Shimadzu VP series HPLC and Shimadzu Class-VPTM chromatography data systems were used. All chromatographic operations were performed at 25 °C. The chromatographic peaks of all standards were confirmed by comparing their retention times and ultraviolet (UV) spectra. A LiChrospher^®^ RP-18e (250 × 4 mm, 5 μm) column (Merck KGaA, Darmstadt, Germany) was used. The separating conditions included mobile phases and gradient program conditions, modified from the description by Saleh et al. [42]. The mobile phase, same as the description by Saleh et al., was 0.5:35:65 phosphoric acid:methanol:water (solvent A) and 0.5:70:30 phosphoric acid:methanol:water (solvent B). The flow rate was modified with 0.8 mL/min. The gradient program conditions were modified as shown in Table 2.

#### 4.3.3. Trolox Equivalent Antioxidant Capacity (TEAC) Assay

TEAC was measured by the ABTS radical scavenging assay. Briefly, the ABTS radical was prepared from 8 mM ABTS solution and 8.4 mM potassium persulfate solution at a ratio of 2:1. After storage in the dark at room temperature (RT) for 12–16 h, the radical solution was further diluted with ethanol to reach an initial absorbance value (0.70 ± 0.05) at 734 nm. One hundred and seventy-five microliters of the diluted ABTS solution were mixed with 25 μL of the four Cirsium species methanolic extract solutions or Trolox standard. The inhibition percentage (I%) of the radical-scavenging capacity was calculated using the following equation: I% = ((A_ABTS_−A_blank_)−(A_s-ABTS_−A_s-blank_))/(A_ABTS_−A_blank_) × 100, where A_ABTS_ is the absorbance of the ABTS solution, A_blank_ is the absorbance of ethanol instead of ABTS, A_s-ABTS_ is the absorbance of the ABTS solution in the presence of the sample, and A_s-blank_ is the absorbance of ethanol in the presence of the sample. The TEAC values are expressed as Trolox equivalents (mmol trolox/g sample) [28].

### 4.4. Statistical Analysis

A one-way analysis of variance (ANOVA) and then Dunnett’s test were applied to data concerning serum biochemical levels, the activities of hepatic antioxidant enzymes, hepatic GSH and MDA levels, hepatic cytokines levels, and the levels of Cu/Zn-SOD, Mn-SOD, and GST expression. Significant differences in all statistical evaluations were calculated using SPSS software (version 22, IBM, Armonk, NY, USA) and *p* values <0.05 were considered to indicate significance.

## 5. Conclusions

From our present results, we suggest that four Cirsium species in Taiwan possess antioxidant and hepatoprotective effects. *C. arisanense* Kitam. possesses the highest antioxidant activity among the four Cirsium species, and its antioxidant activities are closely related to its PPG contents. *C. japonicum* DC. var. *australe* Kitam. possesses the highest hepatoprotective potency against CCl_4_-induced acute liver damage among the four Cirsium species, and these hepatoprotective effects are related to its silibinin diastereomers (α and β) and diosmetin contents. The hepatoprotective mechanism of *C. japonicum* DC. var. *australe* Kitam. against CCl_4_-induced acute liver damage might be involved in restoring the activities and protein expression of the hepatic antioxidant defense system and inhibiting hepatic inflammation to decrease hepatocyte necrosis. In the future, the antioxidative and anti-inflammatory cellular signaling pathway of the hepatoprotective effects against CCl_4_-induced acute liver damage and the hepatoprotective effects of *C. japonicum* DC. var. *australe* Kitam. against chronic liver fibrosis should be conducted.

## Figures and Tables

**Table 1:** The phytoconstituents of methanolic extracts of four Cirsium species.

Samples	Yields (%)	TPs (mg of Catechin/g)	PPGs (mg of Verbascoside/g)	Silydianin	Silibinin α	Silibinin β	Diosmetin
CAH	23.9	109.65 ± 0.57	175.20 ± 5.45	1.23 ± 0.06	2.53 ± 0.01	-	-
CKH	4.5	93.91 ± 1.07	2.30 ± 0.00_0_	-	1.11 ± 0.02	25.14 ± 0.23	-
CJF	10	49.52 ± 2.11	28.64 ± 1.01	3.28 ± 0.04	0.88 ± 0.01	3.74 ± 0.01	8.66 ± 0.06
CH	15.1	44.86 ± 1.77	25.33 ± 1.24	-	0.38 ± 0.01	0.34 ± 0.01	-

TPs: Total phenolics. PPGs: Total phenylpropanoid glycosides. CAH: The aerial part of *C. arisanense* Kitam. CKH: The aerial part of *C. kawakamii* Hayata. CJF: The flower part of *C. japonicum* DC. var. *australe* Kitam. CH: Cirsii Herba. Columns indicate mean ± SD (*n* = 3).

**Table 2:** HPLC gradient program conditions of methanolic extracts of the four Cirsium species.

Time (min)	Solvent A (%)	Solvent B (%)
0–15	90	10
15–25	70	30
25–35	55	45
35–45	35	65
45–50	0	100
50–55	0	100
55–65	90	10

Solvent A: phosphoric acid:methanol:water = 0.5:35:65. Solvent B: phosphoric acid:methanol:water = 0.5:70:30.

## Abbreviations

ABTS	2,2′-Azino-bis(3-ethylbenzothiazoline-6-sulphonic acid)
ALT	Alanine aminotransferase
AST	Aspartate aminotransferase
ANOVA	One-way analysis of variance
CAH	The aerial part of *C. arisanense* Kitam.
CAH-L	Low dose (5 mg/kg) of CAH
CAH-H	High dose (50 mg/kg) of CAH
CH	Cirsii Herba
CH-L	Low dose (5 mg/kg) of CH
CH-H	High dose (50 mg/kg) of CH
CJF	The flower part of *C. japonicum* DC. var. *australe* Kitam.
CJF-L	Low dose (0.5 mg/kg) of CJF
CJF-H	High dose (5 mg/kg) of CJF
CKH	The aerial part of *C. kawakamii* Hayata
CKH-L	Low dose (5 mg/kg) of CKH
CKH-H	High dose (50 mg/kg) of CKH
CMC	Carboxymethylcellulose
Cu/Zn-SOD	Cu/Zn-superoxide dismutase
DAD	Photodiode array detector
DTNB	5,5′-Dithio-bis-(2-nitrobenzoic acid)
GPx	Glutathione peroxidase
GR	Glutathione reductase
GSH	Glutathione
GST	Glutathione *S*-transferase
H&E	Hematoxylin and eosin
IL-1β	Interleukin-1β
MDA	Malondialdehyde
Mn-SOD	Mn-superoxide dismutase
NADPH	β-Nicotinamide adenine dinucleotide phosphate
PPGs	Phenylpropanoid glycosides
ROS	Reactive oxygen species
RT	Room temperature
SOD	Superoxide dismutase
TBA	Thiobarbituric acid
TBARS	Thiobarbituric acid reactive substances
TEAC	Trolox equivalent antioxidant capacity
TNF-α	Tumor necrosis factor-α
TPs	Total phenolics
